# An Unusual Wound Complication Incident to an Operation for Congenital Phimosis

**Published:** 1880-12

**Authors:** S. N. Jordan

**Affiliations:** Columbus, Ga.


ATLANTA
### Medical and Surgical Journal.

Vol. XVIII.] DECEMBER—1880. [No. 9.

### Original Communications.

### AN UNUSUAL WOUND COMPLICATION INCIDENT TO AN OPERATION FOR CONGENITAL PHIMOSIS.

By Dr. S. N. JORDAN, Columbus, Ga.

None of the usual text books mention a possible serious complication following a simple incision for circumcision in phimosis This operation I have performed six times in adults and once in a child, and only in one single instance has it been attended by any trouble in the slightest degree.

The case in question was as follows: Child five years old of strumous diathesis, left leg perceptibly smaller than the right, due to essential paralysis. Patient has a complete phimosis and very long prepuce. He was troubled with frequent micturition.

Under chloroform the prepuce was incised and excised, then the edges of the wound were united by six silk sutures. This wound healed up kindly in eight days, leaving, however, a dog-ear like flap which was removed and wound closed with one stitch In twelve hours the prepuce and scrotal sack began to swell. In forty-eight hours the scrotum was distened to the size of a man's fist. At the same time the penis swelled until it was several times its natural size. The tissues over the pubic bone were likewise distended. In the inginual region an erythematous rash developed, which disappeared after twenty-four hour's duration. The puffing remained in the part about three days, when it began to subside, at first perceptible in the scrotum. The edges of the wound sloughed some, and an ulcer as large as a nickel piece formed on the penis.

The treatment consisted in a poultice of Peruvian bark and charcoal for eight hours, afterward blue ointment was applied over testicles and all of the penis, save the wound and ulcer; these were sprinkled with iodoform. There were no vesicles to be seen, and the testicles did not participate in the inflammation. There was a slight elevation of tempurature until the swelling began to subside.

I think this was a case of hydropi irritations, brought about by the irritation incident to the repetition of the excision of the prepuce. It is well known that from a scratch or other slight mechanical irritation, œdematous conditions are not rare.

This case I record as it clearly demonstrates that the operation for phimosis can be followed by somewhat alarming symptoms, and that, too, by symptoms other than those generally given by the authors whose works are best known.

I will add that the child made a good recovery. The immediate result—a remission in the frequency of micturition—for which the operation was performed, was obtained.

